# T Cell Co-Stimulation: Inhibition of Immunosuppression?

**DOI:** 10.3389/fimmu.2018.00974

**Published:** 2018-05-03

**Authors:** Karl-Gösta Sundqvist

**Affiliations:** Division of Clinical Immunology, Department of Laboratory Medicine, Karolinska Institutet, Clinical Immunology and Transfusion Medicine at Karolinska University Hospital, Stockholm, Sweden

**Keywords:** co-stimulatory molecules, adhesion molecules, motility, cell surface receptor, shedding

## Introduction

Effective T cell-dependent immune responses require an antigen-specific signal through the T cell receptor (TCR) and simultaneous antigen non-specific signaling through a co-stimulatory receptor, whereas antigen signals alone induce anergy. Besides the prototype co-stimulatory receptor CD28, T cells also express a variety of other co-stimulatory receptors (1). However, although co-stimulation is an established crucial mechanism, there are a several generally neglected causes of uncertainty about its role. It is thus difficult to reconcile the fact that co-stimulatory signals are completely independent of TCR signaling (2–9) with a simple coupled enhancing effect on TCR-induced activation and this is reinforced by the abundance of co-stimulatory receptors (1). The uncertainty about the role of co-stimulation as simply stimulatory is further reinforced by the fact that T cell activation in its absence does not generally cause anergy but development of regulatory T (Treg) cells (10–12), and that co-stimulation is not obligatory for T cell activation *per se*. The immense power and lethality of T cell responses induced by certain superagonistic anti-CD28 antibodies and superantigens may also seem inconsistent with the concept that co-stimulation simply delivers an immunostimulatory signal (13, 14).

A different interpretation is suggested by recent findings that some forms of co-stimulation, rather than being stimulatory, inhibits a mechanism preventing TCR-induced activation (15). The studies unveiling this role of co-stimulation were initiated by evidence that T cell motility, adhesion, and activation depend on the large transmembrane cell surface receptor low-density lipoprotein receptor-related protein 1 (LRP1) and its ligand thrombospondin-1 (TSP-1) (16–22). LRP1 consists of an α-chain (515 kDa) containing ligand-binding domains, a β-chain (85 kDa) containing the transmembrane domain and the cytoplasmic tail (23, 24), and has binding sites for more than 40 ligands. TSP1 is a 450-kDa glycoprotein composed of three identical disulfide-linked polypeptide chains that display binding sites for various cell surface receptors (25). Co-stimulation through CD28, integrins, and CXCR4 inhibits a protease mechanism that removes LRP1 from the cell surface and antagonizes TCR-induced activation (15).

## LRP1 Expression and Function in T Cells

Low-density lipoprotein receptor-related protein 1 deficiency is lethal, which complicates analyses of its function and influence on disease development. TSP-1 deficiency in mice results in inflammation in several organs suggesting that TSP-1 has a protective role in inflammation (26, 27). LRP1 is poorly expressed on T cells directly from the blood of healthy individuals and on T cells cultured *in vitro* (15). TSP-1 induces cell surface expression of LRP1 and the chemokines CXCL12 and CCL5 elicit cell surface expression of LRP1 through TSP-1 (17). TSP-1 also binds to LRP1 and promotes integrin-dependent T cell adhesion (19, 21). However, the most prominent cell surface expression and functional impact of LRP1 are unveiled by a broad-spectrum metalloprotease (MMP)/disintegrin-type metalloproteinase (ADAM) inhibitor through inhibition of shedding, indicating that LRP1 is transported to the cell surface and then released by an enzymatic mechanism (15). If cell surface LRP1 is protected by an MMP/ADAM inhibitor, and TSP-1 transport to the cell surface simultaneously is stimulated by cell contact with β1 and β2 integrin ligands, TSP-1 associates heavily with LRP1 (15). This is accompanied by firm integrin-dependent T cell adhesion, which arrests the cells, although not inhibiting motility *per se*, and a potent enhancement of TCR-induced T cell activation. The intracellular TSP-1 induced to appear on the cell surface consists of preformed full-length 170 kDa TSP-1, and 130 and 110 kDa TSP-1 fragments that may represent isoforms and differentially collaborate with LRP1 in the regulation of T cell motility and formation of adhesive contacts (15, 21). Particularly strong evidence on collaboration of LRP1 and TSP-1 in the regulation of T cell adhesion is that adherent cells co-express LRP1 and TSP-1 on the surface, whereas non-adherent cells do not (15, 21). Small interfering RNA (siRNA) silencing experiments support the conclusion that LRP1 and TSP-1 collaborate in the regulation of T cell motility and TCR-induced activation and that TSP-1 induces integrin-dependent adhesion (15). By contrast, siRNA silencing experiments seem to suggest that LRP1 alone is anti-adhesive (20). However, the increased adhesion after silencing of LRP1 probably reflects upregulated cell surface expression of TSP-1 that promotes adhesion (19, 21). One reason for this may be that absence of LRP1 triggers a feedback mechanism to bring TSP-1 to the cell surface to stimulate LRP1 expression (18). Another explanation may be that absence of LRP1 prevents loss of TSP-1 through the LRP1-targeted shedding mechanism. TSP-1 seems to appear on the cell surface alone and then associates with LRP1, and this upregulated TSP-1 expression through complexing to LRP1 seems critical for adhesion (15, 20, 21).

Disappearance of LRP1 from the T cell surface hence maintains a constant autocrine suppression of integrin-dependent adhesion and TCR-induced activation. This may prevent persistent adhesive contacts, excessive and adverse T cell activation, and immune responses in the absence of immune checkpoint proteins and Treg cells. The action of these established immunosuppressive elements is hence restricted by dependence on previous activation, capacity of Treg cells to suppress and compete with conventional T cells, and on sensitivity of their target cells (28–31). The LRP1-targeted immunosuppressive mechanism may also prevent adverse activating signals on T cells by LRP1-binding factors, such as heat-shock proteins (32).

## LRP1 and TSP-1: Tools for Receptor Communication in *cis*, Integration of Signaling Pathways, and Metabolic Reprogramming

A rather plausible explanation why surface-expressed LRP1, in collaboration with endogenous TSP-1, can regulate motility, adhesion, and T cell activation (15–22) is that LRP1 controls and integrates multiple signaling molecules and their pathways (23, 24). LRP1 and TSP-1 also regulate motility and adhesion through receptor communication in *cis* within the same plasma membrane, which may contribute to integrate cell surface receptors and signaling pathways (17, 19, 33). An additional explanation for the influence of LRP1 and TSP-1 on multiple functions is that metabolic reprogramming plays a key role for T cell responses to antigen and other contexts, and LRP1 expression exerts a major impact on cells though control of sugar transport and lipid metabolism (34–36). Activation-induced upregulation of LRP1 and TSP-1 on T cells (22) may thus be important for the altered metabolic requirements by activation. The conclusion that LRP1 plays a key role for T cell regulation through control of cell signaling is supported by the demonstration that motility in T lymphocytes depends on the Janus kinase signal transducer and activator of transcription and phosphatidylinositol 3-kinase (PI3K) pathways (17, 20), the activation of which depends on LRP1 (37, 38). The evidence that LRP1 controls motility and adhesion in T cells (15, 18, 20, 21), is consistent with results obtained using Schwann cells indicating that LRP1 controls adhesion and motility through Rac1 and RhoA (39), which regulate actin polymerization in T cells, and are critical for motility and adhesion (40, 41). It is also interesting that several findings suggest a possible involvement of LRP1 and LRP1/TSP-1-dependent receptor communication in *cis* for control of Treg cell functions. Accordingly, receptors for transforming growth factor beta (TGFβ), which are major regulators of Treg cells, are associated with LRP1 and TGFβ is activated by the LRP1 ligand TSP-1 (24, 27, 42). However, LRP1 can be expressed on virtually all cells consistent with its importance for general properties, such as motility and adhesion, and suggesting a role for the function of naïve as well as effector T cells.

## Co-Stimulation Through Inhibition of LRP1-Targeted Immunosuppression

The LRP1-targeted immunosuppression must not prevent protective immune responses against infectious agents. This requires that the T cell activation process inhibits shedding and increases cell surface expression of LRP1. It is intriguing that this is a common consequence of ligation of several co-stimulatory receptors. Ligation of CXCR4, β1 and β2 integrins, and CD28, thus inhibits shedding and upregulates LRP1 expression on T cells, although to a much lesser extent than inhibition of MMP/ADAMs (15). This is consistent with the finding that a broad-spectrum MMP/ADAM inhibitor markedly enhances T cell activation in an alloantigen rejection model (43). In support of the conclusion that ligation of CD28 inhibits shedding and upregulates cell surface expression of LRP1, CTLA-4, which blocks binding of B7 to CD28, prevents LRP1 expression on T cells (22). This indicates that it is co-stimulation, and not antigen peptide-MHC complexes, that inhibits the shedding mechanism and upregulates LRP1. The CD28 co-stimulation-dependent upregulation of LRP1 is independent of TSP-1, whereas co-stimulation through β1 and β2 integrins, and CXCR4, inhibits shedding and upregulates LRP1 expression through TSP-1 (15). This different dependence on TSP-1 is logical, since TSP-1 is proadhesive (19, 21), and CD28 ligation should not be allowed to induce adhesion and arrest of naïve T cells searching for their cognate antigen. This assumption is supported by results showing that CD28 ligation antagonizes adhesive contacts (44).

Inhibition of the LRP1 shedding mechanism through ligation of co-stimulatory receptors probably depends on inhibition of the protease responsible (15). ADAM10 is a protease candidate for this as suggested by the influence of a specific inhibitor. Likely protease inhibitor candidates are tissue inhibitor of metalloproteases (TIMP)-1 and TIMP-3 that are expressed in T cells and inhibit ADAM10. It is interesting, therefore, that TSP-1 upregulates TIMP-1 expression in tumor cells (45) suggesting that the stimulatory effect of TSP-1 on T cell expression of LRP1 also is mediated through TIMP-1. As far as I have been able to determine, there are no reports that CD28 ligation influences TIMP expression. However, a possible relationship between LRP1, TIMP-1, and CD28 is suggested by the fact that CD28 co-stimulation enhances PI3K activity (46), TIMP-1 signals through PI3K (47), and LRP1 is a major activator of PI3K (36, 37). It is also worthy to note that T cell expression of TIMP-1 is increased in experimental inflammatory disease (48), which supports the possibility that CD28 co-stimulation contributes to this increase. CD28 signals through mTOR, Grb2, PDK1, and NF-κB pathways, all of which are dependent on LRP1 (49, 50), and GRB-2 binding to CD28 activates NF-κB (9). CD28 co-stimulation may direct the metabolic reprogramming of T cells responding to antigen through LRP1, since the glucose transporter GLUT4 is associated with LRP1, and glucose transport is a target for CD28 ligation (34, 51).

**Figure 1:**
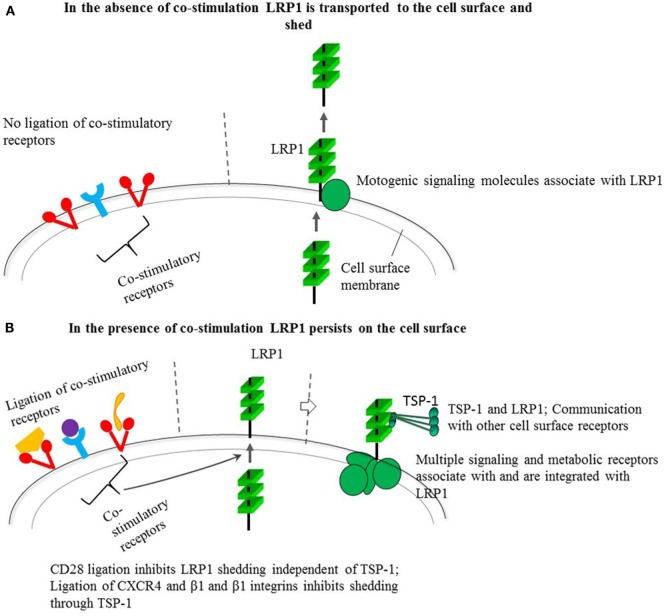
Behavior of low-density lipoprotein receptor-related protein 1 (LRP1) in the absence **(A)** and presence **(B)** of co-stimulation through different receptors and its possible impact on cell signaling through the multiple molecular interactions and connections of LRP1 and its ligand thrombospondin-1 (TSP-1). The constitutive shedding-dependent low cell surface LRP1 as shown in a favors motility, whereas the upregulated level induced by co-receptor ligation by B7, integrin ligands, and CXCL12 also may trigger activating signals through LRP1-dependent expression of signaling and metabolic receptors as well as LRP1-associated TSP-1. TSP-1 binds to cell surface receptors, components of the extracellular matrix, other matricellular proteins, growth factors, cytokines, and proteases (25). Besides its interactions with signaling molecules, as mentioned in the text, LRP1 can interact with multiple different exogenous ligands including α-2-macroglobulin, tissue plasminogen activator, plasminogen activator inhibitor, and apolipoprotein E. Apolipoprotein E is involved in fat metabolism and is produced by macrophages pointing to a possible influence on antigen presentation. It is conceivable that LRP1 and associated TSP-1 in collaboration can communicate with other cell surface receptors besides connecting to or integrating vital pathways for cell signaling or cell metabolism.

The inhibitory effect of co-stimulation on the LRP1-targeted immunosuppressive mechanism, together with the dependence of CD28 signaling on LRP1, that LRP1 integrates signaling (23, 24), and collaborates with TSP-1 (17, 19, 33), have several important implications. Hence, co-stimulation may upregulate cell signaling and receptor communication in *cis* through upregulated cell surface expression of LRP1 and TSP-1 (Figure 1). Antigen peptide-MHC complexes alone may be unable to induce effective immune responses because they do not inhibit the LRP1-targeted immunosuppression. The requirement of co-stimulation for effective T cell activation may reflect that co-stimulation inhibits this suppression. The LRP1-targeted immunosuppression and the antagonistic co-stimulatory pathways may have evolved to combine defense against microbial pathogens with protection against excessive and adverse immune responses.

Further clues to the role of co-stimulation are provided by the fact that direct abrogation of the LRP1-targeted immunosuppression with an MMP/ADAM inhibitor is a more powerful stimulus for T cell adhesion and activation than co-stimulation. This implies that co-stimulatory signals are set not to abrogate the full power of the LRP1-targeted immunosuppression. The power and constant operation of this mechanism both before and after activation suggest that immunosuppression is a prioritized condition of fundamental importance for the organism.

It is reasonable to assume that only a certain part of the many co-stimulatory receptors (1) inhibit the LRP1-targeted immunosuppression. However, the ones so far demonstrated to share this property are molecularly diverse, suggesting that inhibition of the LRP1-targeted immunosuppression may be a common feature also of other co-stimulatory receptors. The interactions of LRP1 and TSP-1 with multiple other molecules may thus endow different cell surface receptors with capacity to induce co-stimulation.

## Conclusion

Formation of adhesive contacts and TCR-induced activation are antagonized by shedding of LRP1. This may prevent persistent T cell adhesion, allowing the search for cognate antigen and target cells, and may also prevent excessive and adverse immune responses. Some co-stimulatory pathways may have evolved to inhibit this suppressive mechanism.

## Author Contributions

The author confirms being the sole contributor of this work and approved it for publication.

## Conflict of Interest Statement

The author declares that the research was conducted in the absence of any commercial or financial relationships that could be construed as a potential conflict of interest.

## Abbreviations

LRP1, low-density lipoprotein receptor-related protein 1; TCR, T cell receptor; Treg, regulatory T; TSP-1, thrombospondin-1; JAK STAT, Janus kinase signal transducer and activator of transcription; PI3K, phosphatidylinositol 3-kinase; TGFβ, transforming growth factor beta, MMP, metalloprotease; ADAMs, disintegrin-type metalloproteinases; siRNA, small interfering RNA; TIMP, tissue inhibitor of metalloproteases; mTOR, mammalian target of rapamycin.
